# Report of a clinical and laboratory management of cell therapy for knee cartilage in the face of mycoplasma contamination

**DOI:** 10.31744/einstein_journal/2022RC6918

**Published:** 2022-06-14

**Authors:** Alessandro Rozim Zorzi, Eliane Antonioli, Juliana Aparecida Preto de Godoy, Oswaldo Keith Okamoto, Andrea Tiemi Kondo, José Mauro Kutner, Camila Cohen Kaleka, Moisés Cohen, Mario Ferretti

**Affiliations:** 1 Hospital Israelita Albert Einstein São Paulo SP Brazil Hospital Israelita Albert Einstein, São Paulo, SP, Brazil.

**Keywords:** Knee, Cartilage, Cell-and tissue-based therapy, Chondrocytes, Mycoplasma, Cell culture techniques

## Abstract

To describe a case of autologous chondrocyte implantation after cell culture contamination by *Mycoplasma pneumoniae* and the measures taken to successfully complete cell therapy in a patient with focal chondral lesion. A 45-year-old male patient, complaining of chronic pain on the knee and no history of trauma. He had a chondral lesion in the trochlear region of the femur and clinical tests compatible with pain in the anterior compartment of the knee. Conservative treatment failed to alleviate symptoms. Surgical treatment was indicated, but due to the size of the lesion, membrane-assisted autologous chondrocyte implantation was the technique of choice. Cartilage biopsies were collected from the intercondylar region of the distal femur. After isolation, chondrocytes were expanded *ex vivo* in a trained laboratory, for three weeks, and seeded onto a commercially available collagen membrane prior to implantation in the knee. Two days before surgery, a cell culture sample tested positive for *Mycoplasma pneumoniae*. The source of contamination was found to be autologous blood serum, extracted from the patient´s peripheral vein, and used to supplement the cell culture medium. After treating the patient with antibiotics, all procedures were repeated and the new final cell product, free from contaminants, was successfully implanted. We discuss the strategies available to deal with this situation, and describe the results of this particular case, which led to modifications in the autologous chondrocyte implant protocol.

## INTRODUCTION

Autologous chondrocyte implantation seeded in collagen membrane is the gold standard surgical technique to treat focal chondral lesion of the knee. It is especially indicated for larger than 2cm^2^ lesions or those located in the trochlear region.^(1,2)^ Chondrocytes, the main ingredient of this technique, are obtained from the patient’s own cartilage through an arthroscopy-assisted biopsy. Since the number of cells obtained in the biopsy is insufficient to obtain the desired therapeutic effect, a 3 to 4-week period of *ex vivo* cell expansion is required before the definitive implantation.^(1-4)^

Mycoplasmas are the smallest free-living microorganisms in nature. They belong to the family *Mycoplasmataceae.*^(5)^ Their size is intermediate between bacteria and viruses; they differ from the former by lack of a cell wall, and from the latter by the fact they grow in cell-free media. The lack of cell wall allows direct contact with host cells, leading to exchange of cell elements.^(6)^ Mycoplasma contamination is a major concern for *in vitro* cell culture for several reasons: due to its small size, it is able to pass through sterilizing filters that would otherwise prevent contamination; the absence of a cell wall makes it resistant to many antibiotics; it causes altered metabolism, morphological changes, and decreased cell viability; it changes the expression of eukaryotic cell genes, potentially altering cell culture performance and final cell therapy product quality.^(5)^Therefore, mycoplasma detection and elimination are necessary for *in vitro* cell culture.^(5-12)^

Cell therapy laboratories are required to operate under strict good manufacturing practice conditions to minimize possible mycoplasma contamination. Nonetheless, occasional mycoplasma contamination of cell therapy products may also derive from the biological sample donor.^(5)^ We report a rare case of autologous chondrocyte implantation seeded in collagen membrane product contaminated by *Mycoplasma pneumoniae,* and the procedures adopted to overcome it, which eventually resulted in a successful autologous chondrocyte implantation therapy.

## CASE REPORT

A healthy 45-year-old man complained of right knee pain for over one year, with no history of trauma. The onset of symptoms was insidious, and the pain gradually worsened, until it began to hinder the recreational practice of his favorite sport, soccer. Six months before, he had undergone surgery on the left knee to treat another focal chondral lesion, at another hospital.

**Figure 1:**
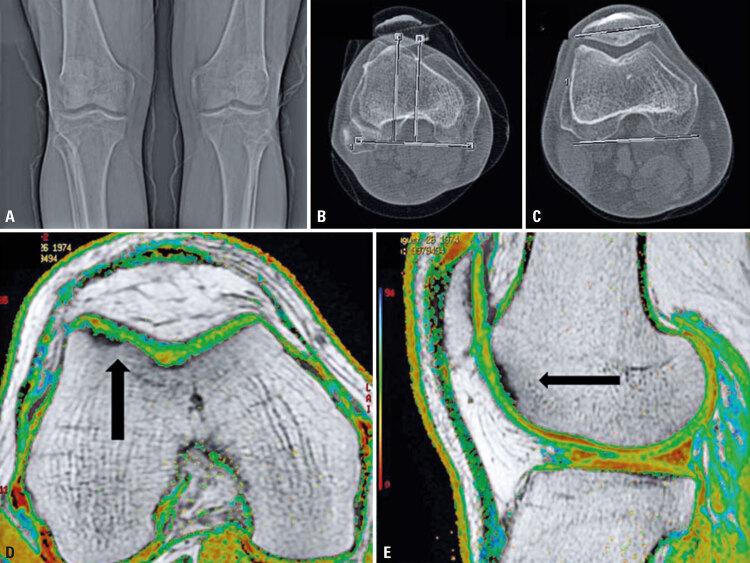
Right knee images before surgery. (A) X-ray with no sign of osteoarthritis; (B) Computed tomography scan with normal TT-TG index (14mm) and C) patellar tilt (7 degrees); (D) T2-weighted magnetic resonance image in the axial plane with focal chondral lesion in the trochlear region (arrow); (E) T2-weighted magnetic resonance image in the sagittal plane view showing the same lesion (arrow)

Physical examination, radiograph and magnetic resonance image of the knee were compatible with an focal chondral lesion in the trochlear region in the right knee (Figure 1).

Due to failure of conservative treatment, surgery with autologous chondrocyte implantation seeded in collagen membrane was indicated. The lesion was confirmed by knee arthroscopy, and biopsy was performed with approximately three grams of cartilage from the femoral intercondylar notch. This tissue was sent to proper facility in the hospital, according to the good manufacturing practice, for isolation and expansion of the chondrocytes.

To avoid using animal products and considering the high cost of xeno-free medium, the local *ex vivo* chondrocyte expansion protocol uses 20% autologous blood serum (AS20%) to supplement the cell culture medium. To ensure greater comfort to patients, the peripheral blood is harvested in the operating room during cartilage biopsy. A total of 200mL of blood was collected in a donation bag without anticoagulant. Patient answered a clinical interview with questions similar to those applied to a blood donor, but since the product is autologous, screening was performed according to Blood Bank guidelines. However, the mycoplasma test was not done before use in cell culture; it was only performed as a release test, according to Brazilian Cell Therapy Guidelines (RDC 214/2018).^(13)^

**Figure 2:**
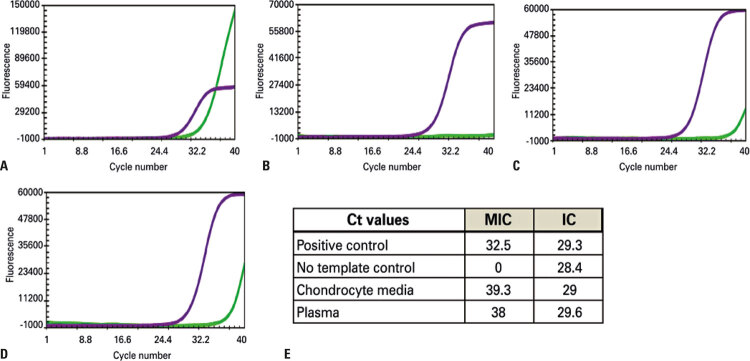
Real-time polymerase chain reaction for *Mycoplasma pneumoniae* from the first culture of chondrocytes derived from patient’s cartilage. (A) Positive control; (B) No template control; (C) Media obtained from chondrocytes culture; (D) Plasma; (E) Cycle threshold values. Purple curve: internal control and green curve: mycoplasma

After three weeks of cell expansion, a slow cumulative cell population doubling level was detected (Figure 2), but it was possible to reach the necessary dose of 6 x 10^6^ cells per cm^2^ on the membrane (Chondro-Gide, Geistilich).

Ct: cycle threshold; MIC: mycoplasma; IC: internal control.

**Figure 3:**
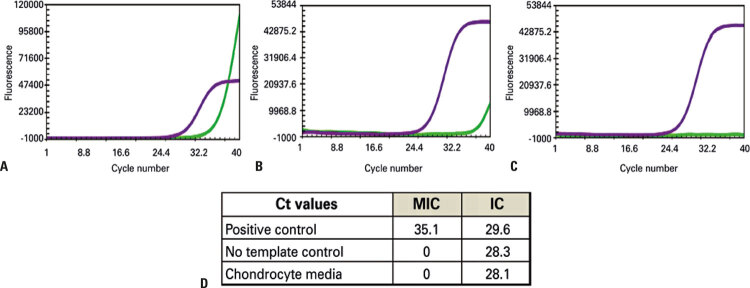
Real-time polymerase chain reaction for *Mycoplasma pneumoniae* from the second culture of chondrocytes derived from patient’s cartilage. (A) Positive control; (B) No template control; (C) Media obtained from chondrocytes culture; (D) Cycle threshold values. Purple curve: internal control, green curve: mycoplasma

Two days before cell product release for surgery, quality tests indicated that microbiological cultures were negative, cell viability was over 90%, and the karyotype had no abnormalities. However, reverse-transcriptase polymerase chain reaction (RT-PCR) was positive for *Mycoplasma pneumoniae* (Figure 3).

Ct: cycle threshold; MIC: mycoplasma; IC: internal control.

In view of the lack of guidelines on how to proceed in this situation, it was decided to discard the material. The surgery was cancelled, and the patient was referred to an infectious disease physician. Serological test was positive for IgM (851U/mL) and IgG (543U/mL). Patient recalled he had symptoms of cold on the day of the biopsy. Polymerase chain reaction analysis of the cell culture medium and plasma showed that the source of cell contamination was autologous serum (Figure 3). The patient was treated with clarithromycin 500mg, bid, for 14 days. The infectious disease physician who followed the case considered it unnecessary to perform new serologies after the treatment, because the patient was completely asymptomatic. The patient was followed up clinically through inquiries about respiratory symptoms and clinical examination of the respiratory system. Postoperative blood tests and chest imaging were not performed. The patient did not present respiratory complaints or alterations in pulmonary auscultation during the entire monthly follow-up until completing six months, and then quarterly, until completing one year.

The autologous chondrocyte implantation seeded in collagen membrane protocol in the institution was modified because of this episode. Blood collection started to be performed before, with RT-PCR test for mycoplasma before use in cell culture.

**Figure 4:**
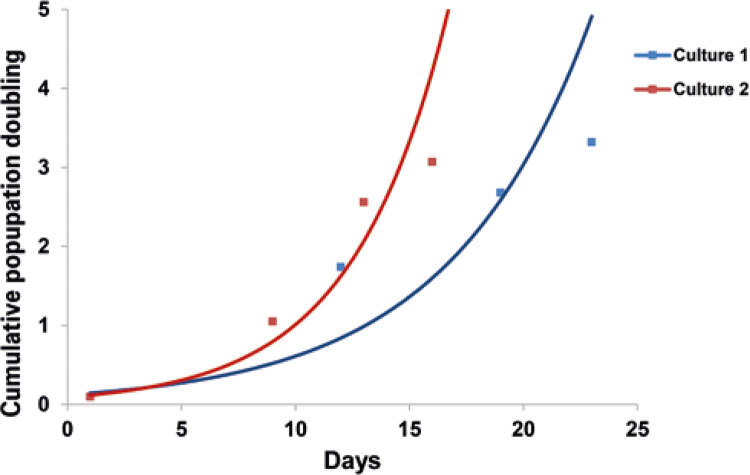
Cumulative population doubling level of patient-derived chondrocyte cultures. Population doubling level was calculated using the following formula: PDL (Population doubling level) = log10 (N/N0) x 3.33. Culture 1: first culture of chondrocytes derived from patient’s cartilage; Culture 2: second culture of chondrocytes derived from cartilage of the same patient. Culture 1 was found contaminated with mycoplasma and culture 2 was mycoplasma-free

In February 2019, a new blood collection was performed in the same participant and the RT-PCR test did not detect *Mycoplasma pneumoniae.* And it was neither detected in cell culture media (Figure 4).

A week later, the patient was subjected to another knee biopsy and the ensuing chondrocyte culture showed an accelerated cell growth, compared with the growth rate of the previous contaminated cell culture (Figure 2). Matrix-assisted autologous chondrocyte implantation was then successfully carried out. One year after matrix-assisted autologous chondrocyte implantation, the patient was discharged and managed to successfully return to sports.

The Research Ethics Commitee of *Hospital Israelita Albert Einstein* (HIAE) approved the study (CAAE: 7800821.5.0000.0071 – # 4.792.019) and the participant signed an informed consent form.

## DISCUSSION

Autologous serum has been used to enhance cell expansion in cell cultures for autologous chondrocyte implantation.^(3,14,15)^ The advantage of this blood product is its low cost and lower risk of transmitting infectious diseases. Regulatory agencies have recommended avoiding the use of fetal bovine serum and other products derived from animals, both because of the risk of disease transmission and the ethical issue of suffering caused to animals to obtain them. On the other hand, many xeno-free and chemically defined serum-free culture media have appeared on the market, but their price is still prohibitive in less economically developed countries.^(16-20)^

For autologous use, donor is not required to microbiological tests before collection. The tests are done during and for cell release to ensure adequate safety and quality of the cell therapy product. However, we herein described the dilemma caused by the positive finding of *Mycoplasma pneumoniae* in RT-PCR, before the release of the cellular product for implantation in the patient. If the patient had performed the RT-PCR for *Mycoplasma pneumoniae* before cartilage collection, he could have been diagnosed and previously treated, preventing the loss of the cell culture product and autologous chondrocyte implantation seeded in collagen membrane delay.

Since the mycoplasma contamination came from the patient’s blood and not from the laboratory environment, it is plausible to question whether release of the final cell product for autologous implantation in the patient would be deleterious or not. In fact, all other parameters, including karyotype, cell viability and cell counting, were normal. Some authors described methods to decontaminate cell culture *in vitro*. Methods for eliminating mycoplasma from cell cultures include physical, chemical, immunological, and antibiotic-based approaches.^(21)^ However, there is no report on the use of chondrocytes from decontaminated cultures in the clinical setting. Moreover, decontaminating a cell culture with mycoplasma is a very difficult task, with no guarantee of success at the end of the process.

## CONCLUSION

This case report describes our experience with a case of contamination of a chondrocyte culture by *Mycoplasma pneumoniae*, which led to the modification of the institutional protocol to perform the autologous chondrocyte implantation seeded in collagen membrane. Even though it is an autologous product, reverse-transcriptase polymerase chain reaction for *Mycoplasma pneumoniae* was adopted for all patients before the collection of the material for culture.
